# Being *in Place*: A Multimodal Analysis of the Contribution of the Patient's Companion to “First Time” Oncological Visits

**DOI:** 10.3389/fpsyg.2021.664747

**Published:** 2021-06-03

**Authors:** Marilena Fatigante, Cristina Zucchermaglio, Francesca Alby

**Affiliations:** Department of Social and Developmental Psychology, Sapienza University of Rome, Rome, Italy

**Keywords:** doctor-patient communication, oncology, companion, conversation analysis, multimodality, participation, Italy

## Abstract

Companions to medical visits have been alternatively viewed as members who “support” or “inhibit” and “interfere” with the doctor-patient interaction. One way of looking at the companions' contribution to medical visits is by coding roles or functions of their communicative behavior. Our paper aims at reconsidering these findings and analyzing how the companion participation is a local and sequential accomplishment, changing from time to time in the consultation. The paper relies upon an overall collection of 58 videorecordings of first oncological visits. Visits were conducted in two different hospitals, one of which a University hospital, and by different oncologists, including both senior professionals and (in the second setting) medical students in oncology. Visits were fully transcribed according to the Jeffersonian conventions and authors examined the transcripts and video according to the methodology of Conversation Analysis. The aim of the paper focused on how patient's companions orient and contribute to the accomplishment of the different aims and activities at different stages of the visit as an institutional speech event. The multimodal analysis of turns and actions (such as, gaze shifts, prosodic modulation, bodily arrangements), and the close examination of the sequential and temporal arrangements of companions' and their co-participants' turns revealed that companions finely attune to the multiparty framework of the encounter and the institutional constraints that govern the oncological first visit. Overall, results show two relevant features: that companions act as to preserve the doctor-patient interaction and to maintain the patient as the most responsible and legitimate agent in the interaction; that companions' contributions are relevant to the activities that sequentially unfold at different stages in the consultation (e.g., history taking, problem presentation, treatment recommendation etc.). The study complements earlier findings on the companion's roles, showing how these are highly mobile, multimodal and multiparty accomplishments, and they are tied to the specific contingencies of the visit. The results solicit to consider the value of multimodal analysis in understanding the complexity of multiparty communication in medical setting, and make it usable also in medical education.

## Introduction

Communicating about cancer poses a huge burden upon patients, due to the high complexity of the information that they have to process (Davis et al., 2002; Han et al., 2011), the intense and potentially frightening emotions that may arise, primarily in the first stages of apprehending the illness (Nail, 2001; Singer, 2018), the variety and relevance of cognitive and decision making processes that have to engage, including the consideration of treatment options, the assessment of benefits and risks, the practical issues related to the beginning of the treatment (Epstein and Street, 2007; Fatigante et al., 2020). In this context, the presence of the patients' companions to the visit can have a strong impact on various aspects of doctor-patient communication, including doctors' and patients' chances to understand each other and/or attune to each other (Pino et al., 2020), and to engage in decision—making (Hubbard et al., 2010; Laidsaar-Powell et al., 2013; Laryionava et al., 2018).

Overall, companions present in the oncological visit—who are most often family members (Lamore et al., 2017) are reported to facilitate the communication between the doctor and the patient and provide instrumental and emotional support to the cancer patients (Ellingson, 2002; Del Piccolo et al., 2014). However, they are also reported as being obtrusive, and inhibiting patient's participation, in settings where the patient is elderly or vulnerable, as well as, at advanced or terminal stages of the patient's illness (Mazer et al., 2014; Pino and Parry, 2019). Relevant to our investigation, is the mention that existing studies on the topic mostly rely upon the ascription of specific individual actions and behaviors of the companions to pre-assigned role categories in coding systems (cf. Street and Gordon, 2008). Verbal behavior and, particularly, self-initiating moves such as, questions, are taken as the primary indicator of their participation (cf. Street and Gordon, 2008; Del Piccolo et al., 2014); less attention is dedicated to the way they engage—and are engaged by their co-participants- through other communicative modalities (e.g., by gaze, gestures, and actions). Further, no distinction is made with regards the moment in the visit where the companion intervenes, overlooking that the medical visit is a sequential, institutional event (Drew and Heritage, 1992) that temporally and orderly unfold through multiple stages (Robinson and Stivers, 2001; Robinson, 2003).

Our work adds to existing literature (Ellingson, 2002), confirming that companions play several functions in support of the patient throughout the visit. However, our study uniquely contributes to this field of research by showing that “roles” are in fact highly mobile accomplishments, subject to the co-participants' responses and ratification; further, we show that the companions' (either discourse and bodily) moves are deeply tied to the specific stages and institutional aims of the visit, and it is only in light of the specific aims and constraints of the visit's stage that the companions' (as well as the other participants') actions can be relevantly interpreted.

We take the example from the oncological setting as an opportunity to indicate that the close analysis of participants' publicly visible and reflexive *actions* in talk (including not only discourse but gaze, gestures, material arrangements of artifacts and tools available to them) is an extremely rich and viable methodology in psychological research interested in the study of communication in sensitive environments.

## Background

There exist extensive evidences that family members, friends, or acquaintances who accompany the patients at the medical visit facilitate the communication between the doctor and the patient and overall play a supportive role, particularly as complex information are delivered and may be difficult for the patient to comprehend (Laidsaar-Powell et al., 2016).

Companions appear to be involved more with elderly patients, patients with increased needs (such as, in pain or in advanced chronic stage of the illness) (Clayman et al., 2005; Ishikawa et al., 2005; Wolff and Roter, 2008; Jansen et al., 2010; Legare et al., 2014; Wolff et al., 2017) or minority patients (Mitchell et al., 2019).

In a 2002 article on interdisciplinary oncological visits with elderly patients, Ellingson identified several roles of the companions, including: aiding in memory, providing emotional support, transcribing information for the patient, aiding in decision making, providing companionship, providing elaboration and context of the patient's response, advocating reasons for patient, and interpreting the doctors' words for the patient.

Other studies indicated that the presence of the companion can generate ambiguities and tensions, due to the fact that companions may sometimes censor the patients' voice, acting as if they were not present (Mazer et al., 2014 call this acting as a pseudo-surrogate of the patient) and display more involvement than desired (by patients) or expected (by doctors) in decision making (Shepherd et al., 2007; Eggly et al., 2013; Laidsaar-Powell et al., 2013, among others).

In cancer visits with newly diagnosed patients, authors have particularly considered the extent to which companions ask questions (Eggly et al., 2006, 2011; Street and Gordon, 2008; Del Piccolo et al., 2014), taken as an indicator of active engagement and support to patients' needs for information.

With particular regards to a collection of Italian visits with (breast cancer) patients, Del Piccolo et al. (2014), reported that most of the (breast cancer) patients' companions in their study helped the patient report or ask for information (e.g., completing the patient's reports, checking or validating the completeness of information), while not inhibiting the patient's involvement in the interaction. In line with what found by Street and Gordon (2008), this study also reported that the companion does not significantly affect the degree of verbal engagement by the patients.

These observational studies, although based on audio- and video- recorded interactions, have subsumed their results *via* coding schema, that is, systems which assign pre-defined values to contributions, namely, utterances or, statements. Basing on pre-assigned categorization of interactional “moves,” authors have, in turn, identified different “roles” to the companion, such as, that of “passive observer,” advocate, partner or “shared role” (Street and Gordon, 2008; Del Piccolo et al., 2014), surrogate or pseudo-surrogate of the patient (Mazer et al., 2014).

Whereas, coding schemas help differentiate among several diverse conducts and positions that may be enacted by the companion in relation to the patient, they suffer from two limitations: (1) they consider the actor's *behavior*, that is, an individual, self-contained unit, almost independent from the sequential context, as the target of analysis and (2) they assign the target behaviors to pre-assigned labels, based on the researcher's hypotheses. As such, they do not capture the interactional details, unfolding *via* verbal and non-verbal resources and the fine coordination among them, which the participants in any ordinary or institutional setting attend to. Focusing on the sequential environment in which participants' turns are allocated (Schegloff, 2007), studies conducted within Conversation Analytic paradigm have demonstrated that the interlocutors' positions in a conversation are highly mobile and always open to negotiation by co-present parties, particularly in a multiparty encounter (Goodwin, 1979, 1984, 1987; Schegloff, 1995, 2000; Goodwin and Goodwin, 2004; among others). Also, to look at how participants coordinate verbal and non-verbal resources is crucial. Despite Mazer et al. (2014) attempted to study the conversational context of the companion's utterances, they only looked at conversational turns (limited to 2), respectively, preceding and following companion statements, and they did not take into account non-verbal, multimodal cues, which have been demonstrated in other contexts as relevant signals for participants to negotiate their initiative at talk (Goodwin, 2000; Mondada, 2007; Stivers, 2008; Ruusuvuori and Peräkylä, 2009).

To date, only few studies have examined companions' initiatives as sequential accomplishments.

Explicitly grounding on Conversation Analytic principles, Pino and Parry (2019) examined the companions' contribution on talk in visits with (terminally ill) patients. In these cases, the sensitivity of topics related to the end-of-life and the pervasive worries that affect both the patients and their significant others, appear to solicit a more active engagement of the companions. The in-depth, sequential analysis conducted by the authors show that, more than binding and simply replacing the patient's opportunity to respond to certain doctor's question, companions' contributions are managed as to sequentially open the relevant conversational slot for the patient to produce a request by herself, i.e., about life—expectancy estimate. Conversation Analysis is also applied by another study by Pino et al. (2020) to analyze healthcare providers' responses to companions' turns in the context of palliative care. Authors show that healthcare providers precisely monitor the sequence of patient- companion's turns in order to avoid to be heard as siding with one or another, and to express a position on an independent, expert basis.

Drawing on the literature examined so far, this article applies a conversation-analytic methodology to the analysis of the contribution of the patient's companion in first oncological visits. These are visits, which occur between cancer patients and oncologists who meet together for the first time, after the patients have already got the cancer diagnosis, and they have also often undergone surgery for that. Basing on the diagnostic assessment, which is routinely reviewed in the visit, these encounters are primarily aimed at presenting and considering the treatment options for the patient, in order to get to a decision.

The study aims at providing an in-depth examination of the companions' participation in the oncological visit. In contrast to coding participants' single *behavior*, we examine how actions are allocated in *sequences* and we analyze participants' turns (either verbal or non-verbal) as the result of complex sequential, multiparty arrangements by all participants. We also take into account the placement of the companions' contribution in the multi-staged structure of the visit. To our knowledge, no study has considered the companions' contribution in relation to the particular structure and the specific institutional tasks and activities that this kind of visit (see Fatigante et al., 2021) involve. Further, we include multimodal cues (such as, gaze and gaze shifts, posture, gestures, modulation of the tone of voice) as essential to indicate how the participants orient to the talk in progress and convey their own understanding of their actions.

## Materials and Methods

### Methodology

The study grounds on the methodological framework of Conversation Analysis (CA). Conversation Analysis is a qualitative method of the analysis of interaction, which uniquely dedicates attention to the sequence of turns and actions, considered as the site for the production of participants' mutual intelligibility (Sacks et al., 1974; Heritage, 1984a).

Accordingly to CA methodology, members' contribution to the talk are only comprehensible within the sequential environment in which the turn was built. Members' contributions are mutually related in minimal sequential units called “adjacency pair,” such as question-answer, greeting pairs, and other sequences such as invitation-response, assessments pairs, formulation—response (confirmation or rejection). In all these conversational pairs, the first pair part instantiates the expectation that the second pair part of a relevant “type” will follow. The absence of the second pair, however possible, will be treated by participants as “relevantly absent,” and can mobilize repair moves (Schegloff et al., 1977) by participants (such as, a re-formulation of the question in the absence of a response) in order to re-establish mutual intelligibility. Not only verbal strings of talk but also, multimodal cues (such as, gaze and gaze shifts, posture, gestures, modulation of the tone of voice) are captured and analyzed by Conversation Analysis as relevant resources by the participants to orient to the talk in progress and reach mutual understanding of their actions. For this reason, transcription is essential. A specific system of notational symbols, named as Jeffersonian system after Gail Jefferson who first implemented it (Jefferson, 2004) ensures that formal aspects of talk production, both intonational and sequential, upon which the analysis is based are made available in the transcripts, constituting the public evidences supporting the validity of the analysis.

The use of Conversation Analysis reveals particularly fruitful as a methodology capable of showing in detail how participants adjust interactional resources as to sophisticatedly pursue different activities and tasks, albeit delicate and complex, in the medical interaction (Heath, 1986; Heritage and Maynard, 2006).

### Data Collection

The collection of videorecordings of first visits took place in the Oncology Departments (Day-Hospital) of two different settings: a medium size hospital (Site 1) and a large University hospital (Site 2) both located in Rome, Italy. The overall corpus counts 58 videorecorded visits, 33 visits collected in Site 1, and 25 collected in Site 2.

Prior to the collection of videorecordings, ethnographic fieldwork was conducted, in order to consider organizational features of the context, e.g., agenda of the visits, availability of the doctors, spatial characteristics of the waiting room, workflow of the visits across the day. Fieldwork lasts overall 2 months, and it comprised taking notes, collection of photographs and formal and informal interviews with the doctors. All this material was also useful to assess appropriate places and times in which to recruit candidate patients and present them the informed consent to the study. Consent was always taken the same day of the patient's appointment with the oncologist.

### Ethics

The study received approval from the Ethical Committee of both hospitals. Written informed consents were obtained from all participants (doctors, patients, and patients' companions). Patients (and companions) were approached and offered the informed consent during their waiting time prior to the visit. Upon the patient's and companion's agreement, a video-camera was positioned in the visiting room. Video-recordings were then safely stored and used for analytical purposes. Images were used, when useful to illuminate how participants' actions and gestures were arranged in relation to talk, as to produce a certain outcome. Due to issue of privacy, images were blurred in order to avoid participants' facial recognition.

### Participants

Participants included 2 senior oncologists (one in each site, with more than 35 years of experience in that specialty) and 4 junior residents in oncology (3 females and one male) in Site 2. In Site 1, all the visits were conducted by the senior oncologist; in Site 2, visits were conducted by resident only (9/25) and by the senior oncologist and one of the resident in the rest of the visits.

Fifty-eight patients and 46 companions participated in the study.

Most patients in the two data corpus are women (80%) who received a breast cancer diagnosis (77%). Their average age (across the two corpora) is 55 years, ranging from 23 as for the youngest to 81 as for the oldest patient. 10/58 were foreign patients, able to comprehend Italian although they did not speak fluently and, in one case, the patient was not able to speak Italian at all.

### Transcription

Videorecorded visits were fully transcribed according to Jeffersonian conventions (Jefferson, 2004), which account for both prosodic and sequential formal aspects of turn production. The transcription of speakers' verbal turns were complemented with the annotation of multimodal aspects (such as, gaze) and bodily actions (Mondada, 2018), co-occurring with the speaker's or co-participants' words. Multimodal markers were taken into account as powerful resources that signal, particularly in multiparty conversations, changes in participation framework (Goodwin, 1984; Goodwin and Goodwin, 2004) and shifts in participants' orientation to the activity in progress (Mondada, 2007), or their mutual understanding and alignments. Names and other references to places (e.g., hospitals) have been modified into fictional ones. Transcription symbols are provided in the Appendix A.

### Data Analysis

The conversation analytic literature now widely available on medical discourse (Heritage and Maynard, 2006) has shown how visits are organized in a particular fashion: they develop accordingly to a series of stages that develop sequentially and orderly, although this order can sometimes admit variations (Robinson and Stivers, 2001; Robinson, 2003; Koenig, 2011; Fatigante et al., 2021). Accordingly, we examined companions' turns in relation to the specific stage of the visit in which they occurred, analyzing whether and how they supported its related aims and activities.

As mentioned, we also dedicated a particular attention to the sequential and temporal management of multimodal cues (such as, shifts in gaze and posture, gestures, modulation of the tone of voice) in the construction of participants' turns and we made available in the transcripts those aspects, which were treated as relevant by the participants to orient to the talk in progress and reach mutual understanding of their actions.

As for the analytic aim of this paper, transcription of each visit was read independently by each author, who sorted out all instances in which the companions contributed to the accomplishment of the different stages of the visits (cf. **Table 2**; as for how stages were identified, see Fatigante et al., 2021). We removed from this analysis one visit only, in which the patient could not speak Italian at all. We considered that the specificity of the companion's role as language broker in this case (however she was not a formal interpreter) made the visit and the participants' arrangements in turn taking much different from the others and required examination in a different paper.

In line with the qualitative methodological perspective adopted, we focused on the sequential development of the conversational excerpts and paid attention to what the companion's turn (either expressed by discourse moves or bodily resources) responded, and what “next” relevant action it originated (Sacks et al., 1974).

## Results

A companion was present in 38 (66%) of the visits of our data corpus. Accompanied visits rate higher in Site 1 (75%), in which the mean age of *all* patients is also higher (60.9 vs. 50.5), while in Site 2 the number of accompanied and unaccompanied visits is similar (52%).

Notably, among patients older than 75 years old (6 in both corpora), 83% were accompanied. As regards the gender of accompanied vs. unaccompanied patients, female cancer patients tend to be accompanied more than their male counterparts.

The overall number of companions is 46, a figure that exceeds the number of patients, indicating that some visits included more than 1 companion. Table 1 describes the relationship that the companions had with the patient.

**Table 1:** Relationship of the companion to the patient.

Family members	34 (73.9%)
Friends/acquaintances	11 (23.9%)
Paid caregiver	1 (2.2%)

Family members included the patient's spouse for the most part (53%), an adult child for a smaller proportion (28%), a sibling and one (or both) parents of the patient.

### The Stages of the Oncological Visit

In order to delineate a few quantitative coordinates of the visit as a spatio temporal communicative event, we first provide some background information about the average length of the visits and its different stages. Visits in the corpus last 27.5 min on average, with a maximum length of 40′ and a minimum of 10′, a feature that varied in relation to the time pressure that participant oncologists experienced in the specific day of the data collection (apprehended by the researchers' field notes and participant observation). Daily timetables filled with first oncological appointments generally spanned between 7.30 a.m. and 1.30 p.m. and they included 7 visits per day on average, a number that sometimes varied, to reach up to 12 visits.

As regards the structural organization of the oncology first visits, prior work on this data corpus has delineated different stages (Zucchermaglio et al., 2016; Fatigante et al., 2021), each aimed at performing a different and institutional activity of the visit, and thus also implying a different opportunities, rights, and responsibilities (or, *status*) of participation (e.g., answering doctor's questions vs. listening to his explanations; cf. Heritage and Maynard, 2006).

In the table below we provide a brief overview of each stage.

Parentheses {} indicate that included stages are not always present.

Previous analyses (Fatigante et al., 2021) have also evidenced the relative length of each stage in this visit. So, we mention that the longest, most prominent stage in this visit is the *Outline of future actions* (19% of the visit total length), immediately followed by the *Treatment recommendation* stage (18%) and the *Cancer diagnostic assessment* (14%). The stage of *Cancer problem presentation* stands for the 13% of the visit total length, with the other stages rating almost equally (*Openings* 9%, *History taking* 8%, *Closings* 8%; when present, *Physical examination* rates 5%).

These percentages inform us that the most significant activities in this kind of visit are those, in which the oncologist delivers information, explanations, recommendations and advice to the patients and companions. This feature would support the evidence, gathered since pioneering research on medical interaction (Hall et al., 1987; Bensing and Dronkers, 1992; Emanuel and Emanuel, 1992), for which the instrumental dimension of talk exceeds the socio-emotional one in this kind of setting (cf. also Eide et al., 2003).

Given this picture, which would see the doctor mainly providing information, and the patient (correspondingly) in the position of his main addressee, what the companion can contribute to the development of such an event, and to the different activities that unfold therein?

The excerpts that follow were chosen and selected as particularly clear illustrations of the ways in which the companion contributes differently to the unfolding of the sequence of activities of the visit (see Table 2). We will now provide some examples extracted from each different stage of the visit, which are particularly representative of the strategies, carried out by means of verbal and non-verbal resources, used by the companions to engage in conversation.

**Table 2:** Stages of the oncological visit (Fatigante et al., 2021).

**Stage**	**Definition**
Openings	It includes greeting sequences, sequences of small talk that bridge the participants' official entrance into the business of the visit, followed by identification sequences (such as, the request and registration of the patient's name and address). It is routinely accompanied by the opening and writing of the patient's record.
History taking	It includes the oncologist's activity of questioning regarding the clinical history of the patient (including present and past illnesses, surgical interventions, current pharmacological treatments, etc.), beyond the recent cancer diagnosis. It is relevant in order for the oncologist to assess cancer comorbidities, useful to plan a treatment recommendation that has no harmful consequences for that particular patient (Zucchermaglio et al., 2016; Pino et al., 2021)
Cancer problem presentation	This stage includes the patient's description and narrative regarding the current cancer problem: when it has been discovered, how, when the patient has undergone surgery etc. It is quite short in Site 1, where the oncologist only asks how the patient discovered it and then asks the patient to see the documents; in Site 2, the patients and companions are left more time to build narratives of the realization of the tumor and events that follow that, which can develop across several turns
Cancer diagnostic assessment	Also corresponding for the most part to what in oncology is referred to as the “staging” of the cancer, the diagnostic assessment stage includes the examination of tests brought by the patient (mammography, ultrasounds, surgical reports, and primarily the histological exam) and the explanations given to the patient about the figures and tests
Treatment recommendation	It comprises the presentation and discussion about the treatment options. It includes even lengthy and highly complex explanations about the risks and benefits of the treatment. It also sometimes, but not routinely, include reference to collateral effects and prognostic assessments.
{Physical examination}	Physical examination may occur either to aid in the diagnosis of the cancer size, location or progression or to assess the post-surgical scar on patient's body
Outline of future actions	It comprises the oncologist's verbal recommendation and written prescriptions of next appointments, exams; it also includes instructions about the practical management of the illness (e.g., changes in work agenda, whom to call if the patient feel sick after the treatment etc.)
Closings	It is marked by the participants' orientation to the closing of the official business of the visit, such as, closing, removing documents from the table and folding them, acknowledgments, greeting sequences

### Companion's Participation in Opening the Visit: Engaging in Small (Sociable) Talk

However routinized, openings in interaction imply a complex coordination by interactants (Schegloff, 1968; Duranti, 1997): particularly in institutional exchanges such as a medical visit, members have to concurrently and timely manage their entrance onto the official business of the encounter, thus rapidly traversing each other's self-presentation. Despite this, we have found that visit openings do not only include greetings but also small talk sequences (Laver, 1975; Coupland, 2000; Holmes, 2000). These are sequences, often found in correspondence to “boundaries” of the interaction (Laver, 1975), which are not necessary to the instrumental task of the interaction, but they help participants to establish a common ground and ultimately test that they will be mutually cooperative partners (Maynard and Hudak, 2008).

In the next excerpt, the oncologist (Site 1) is filling the patient's record with his personal data: he has already asked the patient's name and what his job is. The oncologist's inquiry reveals that the patient works as seller of a coffee company, whose brand the oncologist knows. This originates a sequence of small talk.

The initial configuration of the participants' bodies and gaze is the following (see picture): the oncologist is writing down the patient's data, the patient sits with a folder and his jacket on his lap, the companion sits at the left of the patient, with her jacket on.


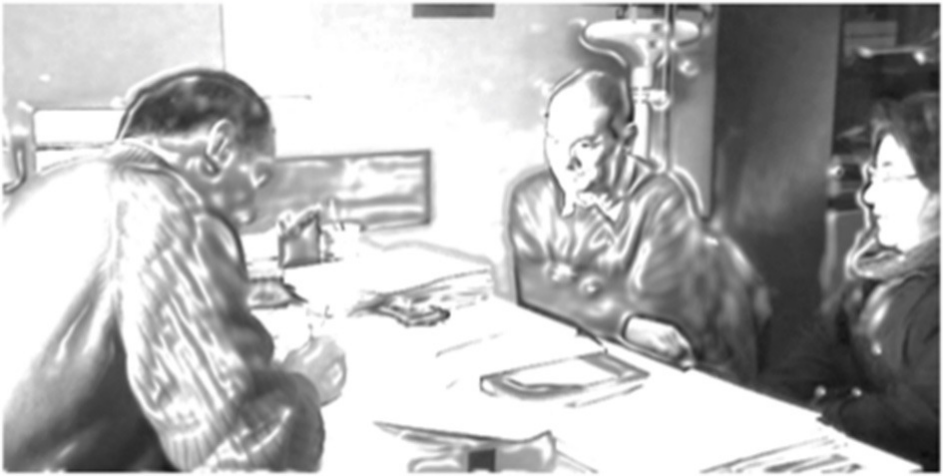



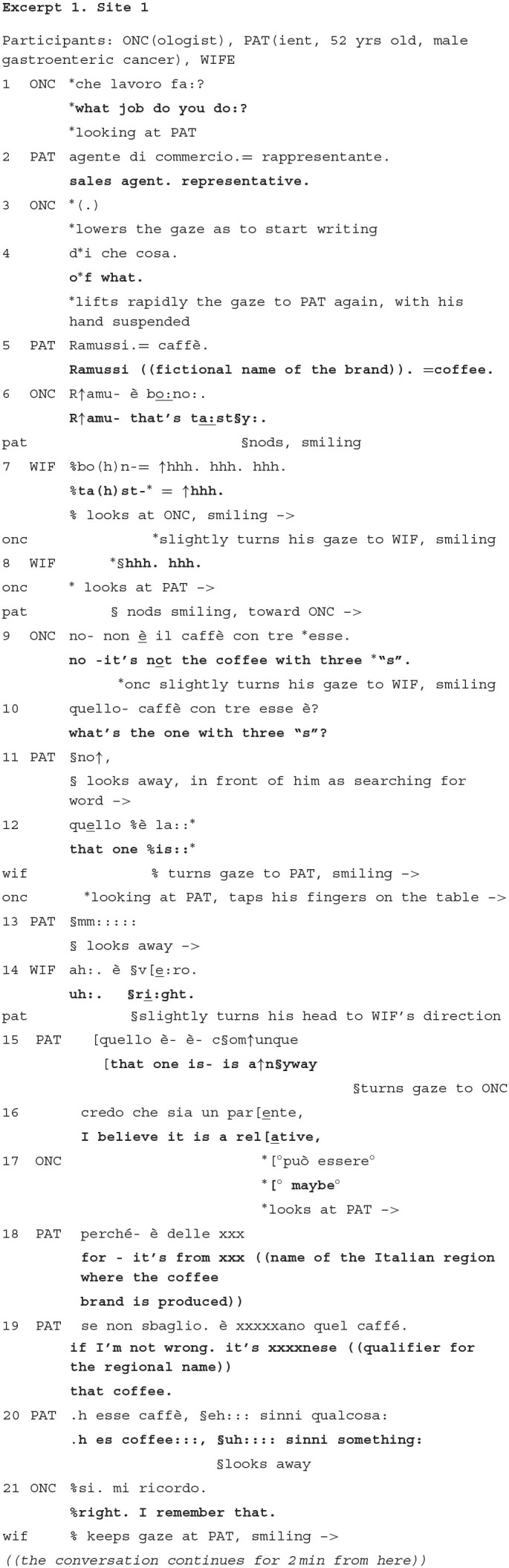


This excerpt shows how the companion *monitors* the oncologist's action since the beginning, while the oncologist writes down the information and continues as the oncologist asks the patient about his job (lines 1–6). The wife enters the conversation, when the oncologist pronounces his assessment of the coffee as “tasty” (line 6): here, the patient's wife immediately lifts her gaze to him, she starts repeating his same assessment and infuses it with laughter, that she continues through subsequent lines. By starting to repeat the doctor's assessment, the wife sides with him (Goodwin and Goodwin, 1992). Furthermore, by initiating and then continuing to laugh (lines 7–8), she self- candidates as an affiliate audience of the doctor's ironic performance, which in turn is indexed by the doctor's choice of the word “bono” (this comes from the Roman jargon and replace the Italian “buono,” meaning “good). By using that term, the doctor is trespassing his identity from the institutional one, of oncologist, to an informal one of a common inhabitant and speaker of Rome (see Ochs and Schieffelin, 1989, for how language indexes geographical provenience). So doing, the patient's wife aligns with the doctor twice: agreeing with his assessment and affiliating with the ironic key implicit in his formulation (on laughter and affiliation, cf. Jefferson et al., 1987; Glenn, 2003).

From the positions taken by the participants relatively to the videocamera, we cannot make sure whether the oncologist is “responding” to the wife's turn. Yet, it is visible that he slightly moves his head toward her (corresponding to line 7), thus indicating that he acknowledges her initiative.

In the subsequent turns, the oncologist continues to question the patient's “expertise” upon the coffee brand, while the companion takes the position of audience to the talk in progress. She turns to her husband as he manifests uncertainty in the word searching (line 12), and she only utters a formulation later on (line 14: “*uh: right*”): this, tough, does not impose any constraint upon the others' contributions. By acknowledging the evidence, recalled by the doctor, that another brand exists, which might be confused with the one they are discussing, she communicates that she is fully “on topic,” but she does not recruit attention by any of the participants.

Despite this, her presence is visibly acknowledged and used by the patient. Overlapping with the wife's intervention (line 14), the patient turns his gaze in her direction.

In a 1987 remarkable paper “Forgetfulness as an interactive resource,” Charles Goodwin showed that participants engaging in word search use gaze as a “framing device” capable of converting what would otherwise be a private thinking activity onto a “social activity, one that parties other than the speaker can actively participate in” (p. 118). Something similar happens here. Yet, here the patient does not fully complete the action of gazing *at* her wife. His gaze remains mid-way, something, that allow him to maintain availability to the oncologist only. On the other hand, the wife restrains her participation in such a way, as to not inhibit the interaction flow between the patient and the doctor.

However, small talk sequences may appear quite inessential to the institutional unfolding of the main tasks of the visit, their analysis helps to highlight and anticipate what will reveal as the main feature exhibited by the companions' contribution in the visits of our corpus: the placement of their turns in a position, which manifests their effort to avoid taking the floor and engaging directly in the main stream of interaction with the doctor.

*Via* her almost inaudible, interstitial comments, the companion projects her role as ancillary and “appended” to the current speaker's contribution. We will find examples of this in several other parts of the visit.

We show another excerpt, which has different background features from the previous one, and that, however, leads to similar findings.

The next excerpt occurs at 0′40″ from the patient and companion's entrance in the oncological cabinet. The companion, who is a doctor herself, and the oncologist have just found out that they already met before (in a medical conference) and the companion is telling him some details about the site where she works. As she stops, the oncologist asks:


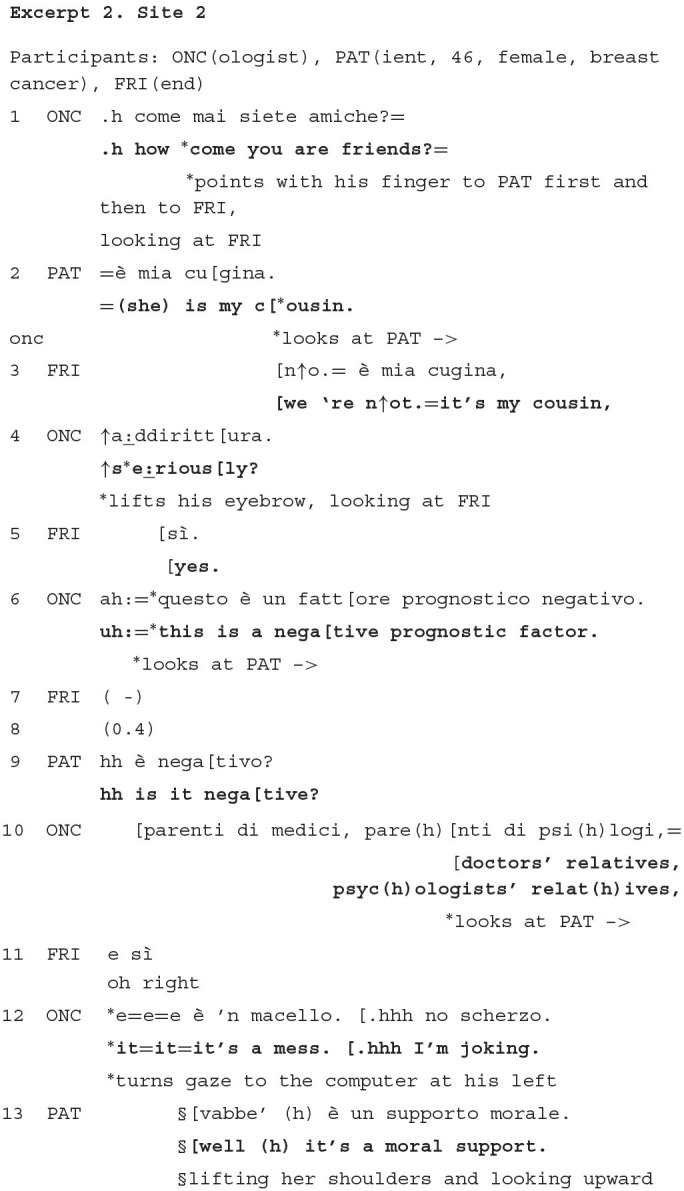


Despite the oncologist recruits both the companion and the patient in asking what is the current relationship between them, and notwithstanding the fact that he was engaging with the companion, and not the patient, in the previous conversation, the companion does not respond to his question. Rather, she leaves the floor to the patient, and only adds her contribution after her, coming as “second.” This poses one of the most common feature of our analyses. The companions' placement of their turns after the patient's one, also when they were fully involved in talk and they would have chance to take the floor.

It is also visible in the junctures of the turns the oncologist's effort to concurrently look at one or the other. At line 4, the oncologist responds looking at the companion, while immediately after (line 6), he shifts gaze and maintains it on the patient, who accounts by telling him that the companion she brought is a “moral support.” Therefore, it is clear from the beginning of the visit that the presence of the companion requires that all participants make efforts to attend to the multiparty framework of this encounter.

### The Companion's Contribution to the History Taking. Monitoring the Patient's Participation

Facilitating information exchange has been documented by studies on companions' and, particularly, family members' contribution to medical visit (Wolff and Roter, 2011). In history taking, this impies that the companions help the patient recall information (Jansen et al., 2010), provide directly information to the doctor, check and validate the patient's report, solve and help repair the doctor-patient's mutual understanding. But how do they do so? We have consistently found in our corpus that, even in cases in which the companions take turn to address the doctor themselves, they pay attention to maintain the patients as the legitimate tellers of their medical history.

Excerpt 3 shows one instance of this practice. Here, the companion contributes in the accomplishment of the activity of recollecting the current medical history of the patient, unfolding through the anamnestic stage. The patient is addressed by the doctor with a question about other non-oncological relevant illnesses.


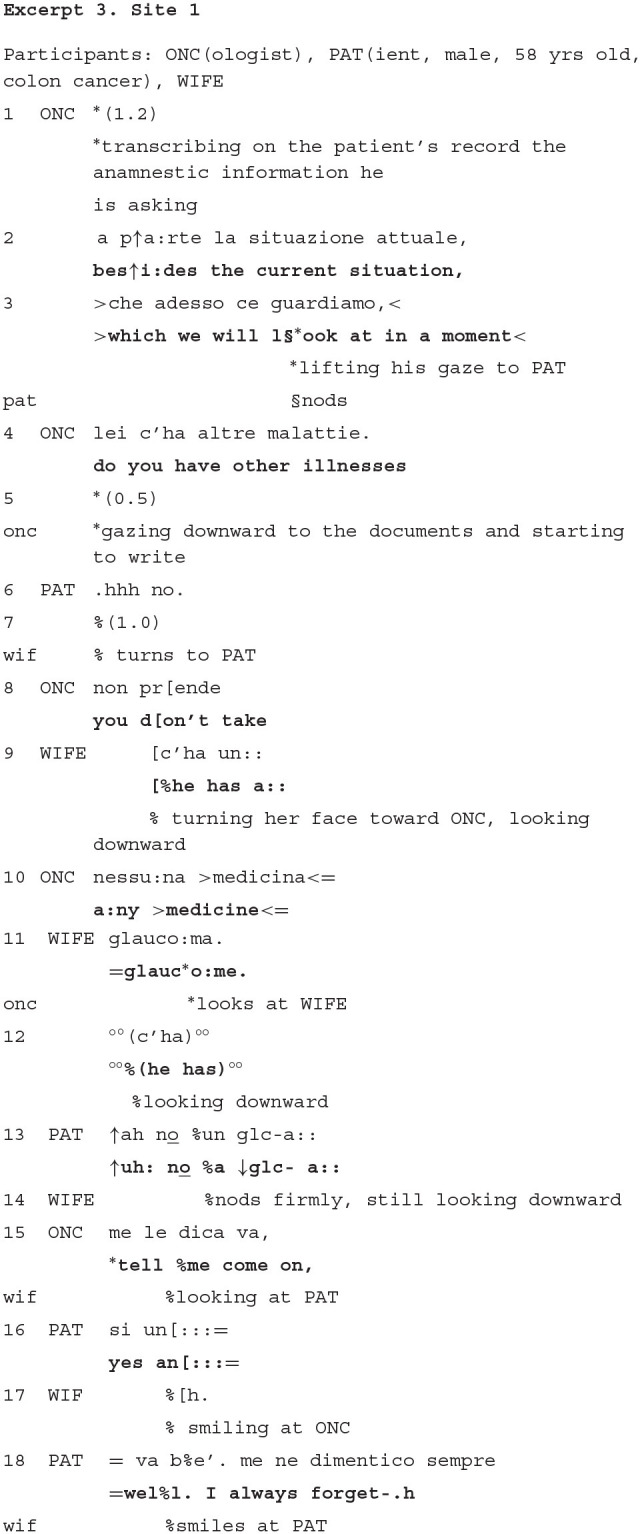


As visible in the transcript, the companion (i.e., wife) gaze—monitors the patient's response (line 7). She only intervenes and prompts the patient's response at line 11, when it is clear that the information provided by the patient, denying the presence of other medical conditions beside the cancer, is about to be registered as valid and permanent (for the oncologist is writing the patient's response on the record).

It might also be noted that the companion revises the patient's answer in such a way (whispering, and avoiding to gaze to any interlocutor in particular, lines 11 and 14) that her turn can be heard as not fully claiming public “visibility,” whereas, it can be captured as a “prompt” by the patient, who produces in fact a change-of-state token (Heritage, 1984b) and starts to repeat (line 17) the name of the illness, incorporating his wife's suggestion.

So doing, while powerfully influencing the content of the response to the doctor's question, the companion has shielded her role of author of the report.

The companion only looks at the oncologist (line 17), after this one has explicitly solicited the patient himself to report information, and by adding an informal token (“come on,” line 15) he ironically treats him as someone who has ostensibly and consciously resisted to tell him (see Craven and Potter, 2010 and Pauletto and Fatigante, 2015 for the use of, respectively, “come on” and “dai” in Italian). By smiling (line 17), the wife conveys her ironic assessment about her husband's inattention and invites the doctor to align with her on this (Jefferson, 1984; Glenn, 2003). The doctor, indeed, maintains his gaze on the patient and will pursue from him, not the companion, the report of the glaucome illness (lines not reported).

In sum, the companion's contribution here was essential in completing and validating anamnestic information. These are usable to assess co-morbidity, which would otherwise be lost, with potential harmful consequences for the patient's cure. Notwithstanding this, it is the patient who is recognized as primary reporter and ratified by both the doctor and the companion as the most relevant addressee and character of the institutional activity of history taking.

We also found evidences of this preference even in cases, where the companion is explicitly ascribed the role of talking “on behalf” of the patient. This is clearly the case, when the patient is not able to speak for himself, due to some impediments related to illness or, to language issue.

The next example provides such a case. The sequence develops during the history taking stage. The patient, a 70 years old man, has undergone a surgery on his tongue and is not so much able to speak fluently. He has identified explicitly the wife as talking on behalf of him at the very beginning of the interaction. Notwithstanding this prior agreement, we observe that: (1) the doctor continues to look at the patient when he asks questions and (2) the wife gazes at the patient as to check for accuracy of information, despite she has access to that.


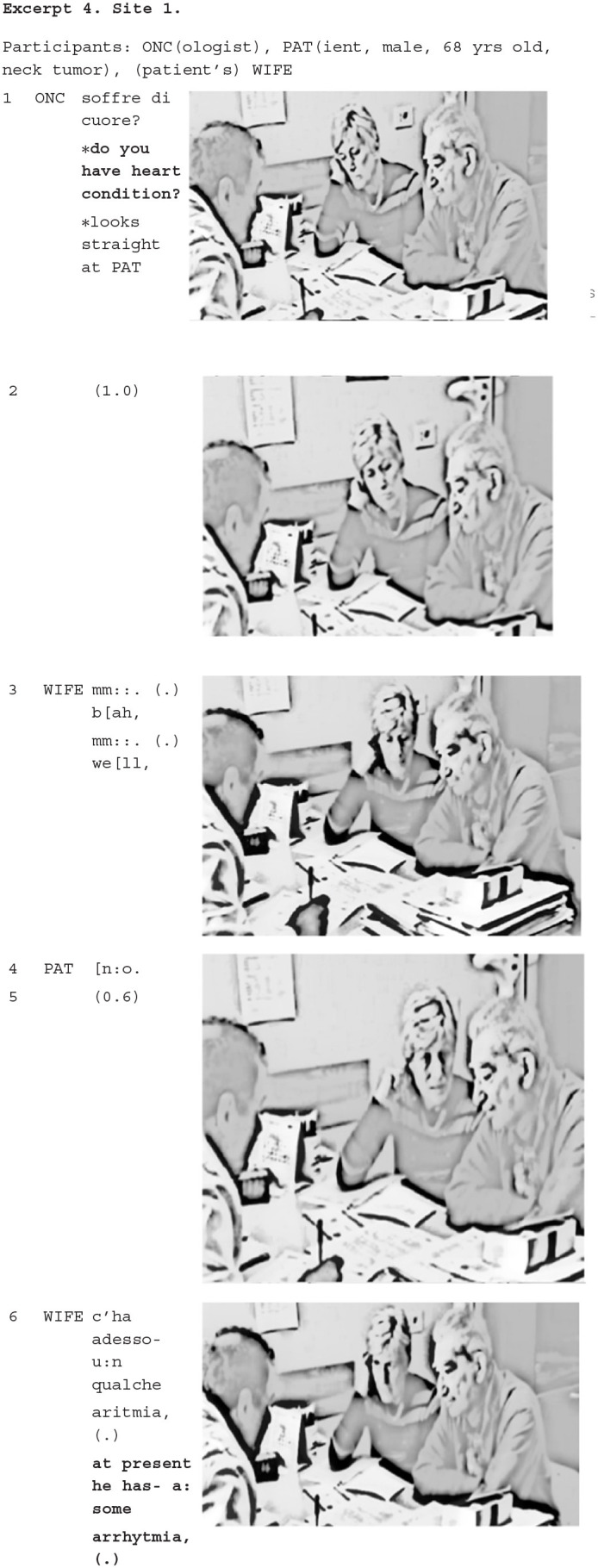


First of all, the doctor gazes at the patient when addressing the question to him. The patient is then considered as the most legitimate teller of his own experience and illness history. Be the one who is entitled to talk on behalf of the patient, it is the wife who starts responding. We see that from line 3, where the wife assumes a partial posture of what Schegloff calls “body torque” (Schegloff, 1998): this is a kind of postural configuration whose main capability is ‘to display engagement with multiple course of action and interactional involvements, and differential ranking of those courses of action and involvement' (p. 536). As a matter of fact, in the course of her response, the wife is concurrently engaged in the interaction with the doctor and the one with her husband, whom she continuously addresses her gaze, as monitoring that he can check and validate the truthfulness of what she tells.

At line 3, the wife's hesitation (mm::.) and the interjection “well,” conveys the sense of a problematic delivery of her response. As she starts vocalizing, the patient slightly bends toward his right (the wife's direction), exhibiting an almost imperceptibly shake of his head, indexing a negative response. He then utters “no” toward his wife, and then looks again at the oncologist. Also, the wife maintains a prolonged look on the patient. As a matter of fact, the wife finally revises the patient's response, by reporting that the patient has in fact “some” arrhythmia.

Yet, there has been a mutual monitoring within the intimate couple, and an orientation to check each other and build upon their mutual knowledge, in order to provide an exhaustive and valid response to the doctor.

What is interesting is that as the definitive response is produced, the wife and the patient co-orient toward the doctor, gazing simultaneously at him.

The response given ultimately to the doctor builds temporally and incrementally upon a mutual coordination of gaze and actions done by both the patient and the companion.

### Negotiating Entitlement to Tell in Cancer Problem Presentation

Cancer problem presentation shares some features with the history taking stage, and related allocation of participants' status: the patient, in fact, is the one who is entitled to report about information s/he has derived from previous visits and from surgery. The main difference is that this stage entails careful examination of the patient's narrative which, in this specialty field, is particularly assisted by medical reports such as, ultrasound, magnetic resonance, surgical reports. These are mentioned and provided as reference throughout the whole narrative about the discovery and diagnosis of the illness.

Excerpt 5 below shows how the companion actively contributes to the accomplishment of the presentation of the cancer problem. Here, the patient's husband revises and validates information provided by the patient, contributing to a precise reconstruction of the tumor first detection.


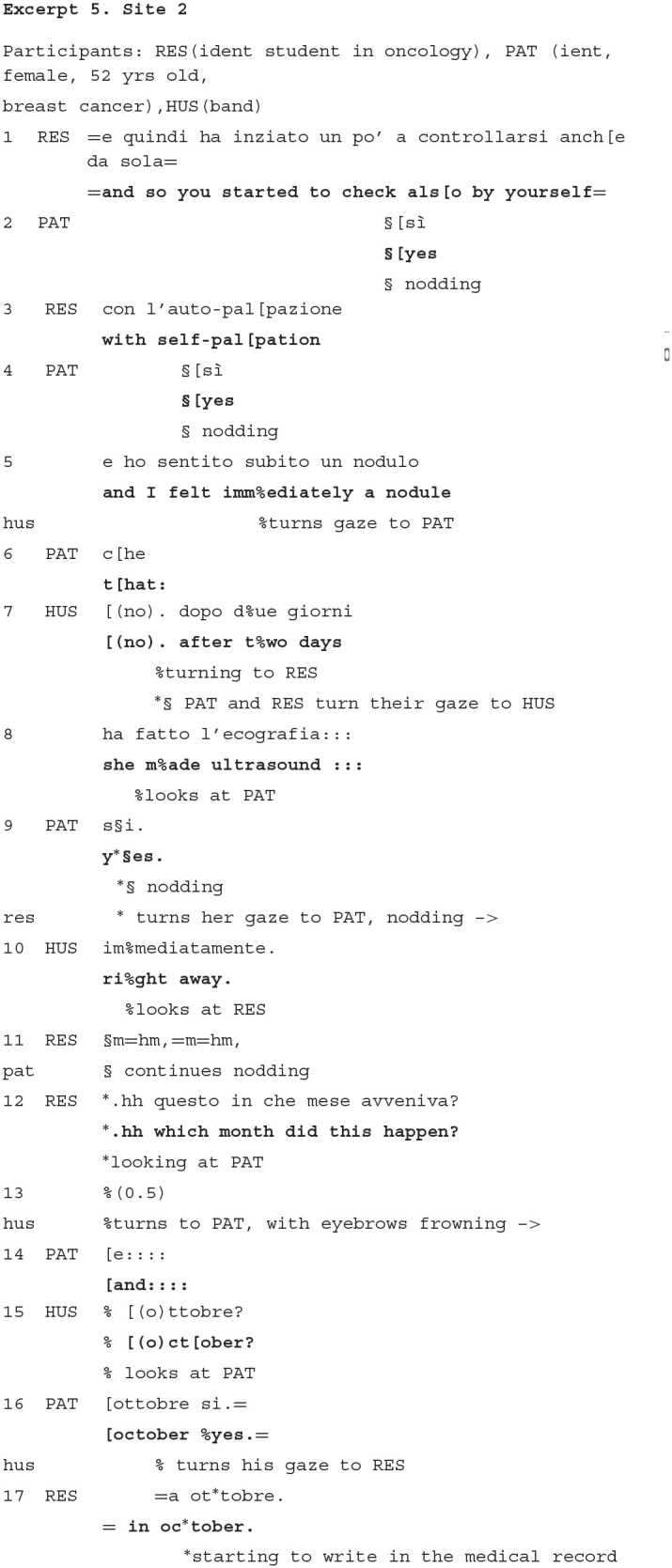


The patient's husband (who is gaze monitoring the patient during her report) enters the conversation in the midst of the patient's turn (line 6), revising her information about the timing of the realization of the tumor first discovered by (subjective) self-palpation and, later, by (objective) ultrasound. Despite he formally “interrupts” the patient, the husband's multimodal displays and particularly, gaze, work as signals that ensure that, throughout the course of all his turns, the patient's alignment and collaboration is constantly checked. Specifically at this regard, the husband turns gaze to the patient when he adds information about ultrasound. The gaze shift manifests the husband's sensitivity to the different knowledge statuses and entitlements of participants in this multiparty encounter (Goodwin, 1979). Throughout his turn addressed to the resident, the companion pursues the patient's validation of the information he is reporting, legitimating her as teller and experiencer of the story. Also, he mentions that the patient did ultrasound “immediately” (line 10): a reference that appears to account for a representation of his wife as a “moral” person who is able to understand the seriousness of a health condition and to engage in reasonable actions to take care of herself with no delay.

As a matter of fact, the resident continues to take the patient as her primary addressee (line 12). This does not discourage the husband to respond (line 15) and collaborate, this way, with the patient helping her to remember and provide accurate information. Yet, by addressing gaze to his wife rather than looking at the resident, he casts his contribution, rather than as a prompt for the patient, a check for validation *from* her, and he ascribes the wife as the one who has the ultimate right to provide a definitive response.

In his attempts to report the events in the right order and as much detailed as they happened, the companion shows to support the institutional aim of this stage, meaning, collecting information about the tumor as much reliably as possible. He does so, while also supporting the patient as a responsible agent in her illness story.

In the following excerpt, the resident is collecting the history of the illness: this routinely includes questioning the patient about when and how she discovered the cancer, how the diagnosis was made and other questions related to surgery, if the meeting is a post-surgical one. A quite high stake imposed over the patient during the problem presentation stage is that the patient needs to provide a reliable telling of the series of events and the evidences that s/he collected prior to the meeting. Particularly when the companions are intimate partners of the patient, such as, spouses, who presumably shared the experience of their illness, they can entitle themselves to supply information asked to the patient, to add, elaborate, or revise them. The problem that surfaces in the example shown, is that the companion and the patient disagree over the timing of an event, which might prove important to tell the oncologist.


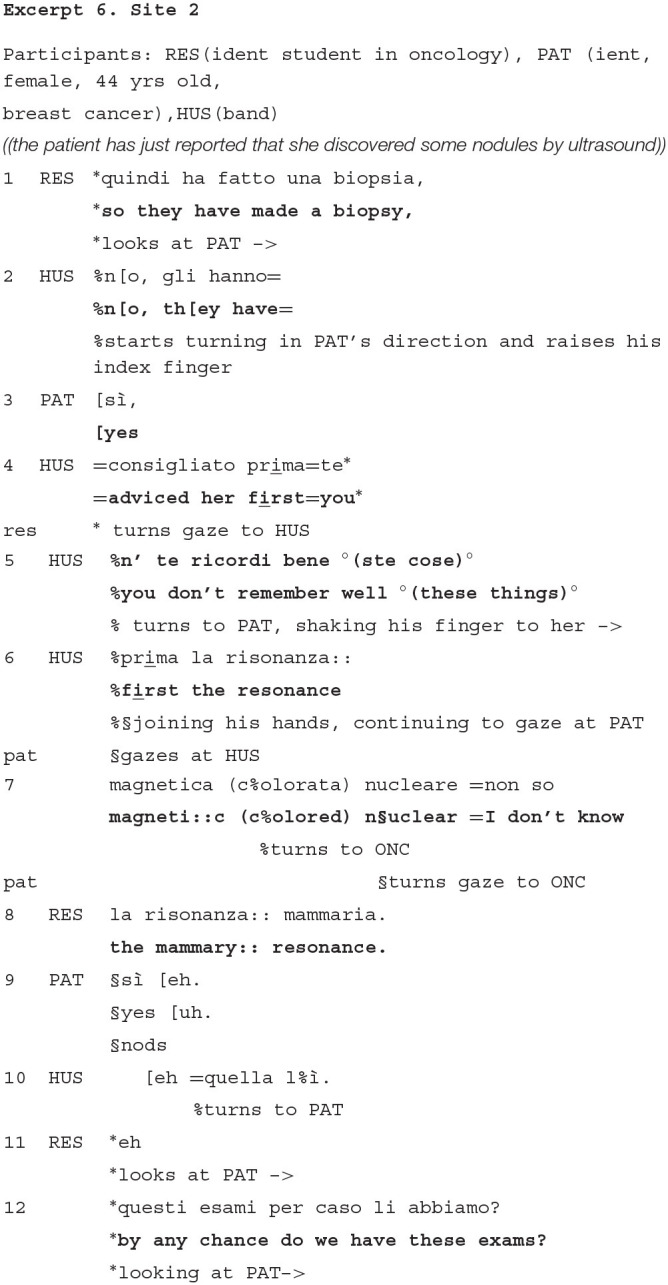


Despite the doctor's question was addressed to the patient, it is the husband who responds first (line 2). In partial overlap with the husband's turn, the patient provides her response (line 3), which confirms the resident's implication, and instead contradicts the husband. Prior to continuing in his telling, the husband turns to the patient and formulates to her that she does not remember well (line 5). What is of analytical interest is that the husband continues to look at the patient throughout his turn, and only turns to the doctor in correspondence with the word search for the exact name of the resonance (line 7). He also frames his turn with an evidential expression (“I don't know”), a stance marker by which he delivers to the doctor the ultimate responsibility and authority to tell about a medical matter.

The gaze shift to the doctor, together with the topical shift (from telling about when the magnetic resonance was exactly done to telling about the specific typology of the resonance) obtains that the husband subsides the disagreement he posed with his wife, with regard the temporal placement of the biopsy in relation to the magnetic resonance. From here on, the official interviewing between doctor and patient is restored: evidences of this are that the companion's response comes after the patient's one (line 10), that the resident only looks at the patient and she puts the sequence with the companion at a close, asking instead the patient about the availability of the documentation.

These analytical findings attest how the companion is capable to orient to the relevant aims proper of this stage, that is, providing information that is as much reliable, relevant and detailed as possible, to the doctor; however, while doing so, he also concurrently orients to not replacing or overshadowing the patient's voice.

### “Sneaking” in Patient's Action During Cancer Diagnostic Assessment

The next series of excerpts show instances where companions help the patient handling the documentation to the oncologist during the cancer diagnostic assessment stage. This stage has been reported a central stage of the whole visit. It entails the careful examination of, particularly, the histological test. This constitutes the bridging document to the recommendation for a treatment option and it implies that the oncologist engages in private diagnostic reasoning (Fatigante et al., 2020), particularly to assess the biological characteristics of the tumor and identify the most suitable treatment.

We have seen that companions closely monitor the patient's activity of handling the documents to the doctor, and sometimes their actions solely prevent a chaotic delivery of the documents.

The next example shows an extract from the consultation with a young patient, who came to the consultation with his wife and two other companions. He meets the doctor for a neck tumor, and he reported, during the history taking, that he was already treated some years before for an Erwing sarcome.


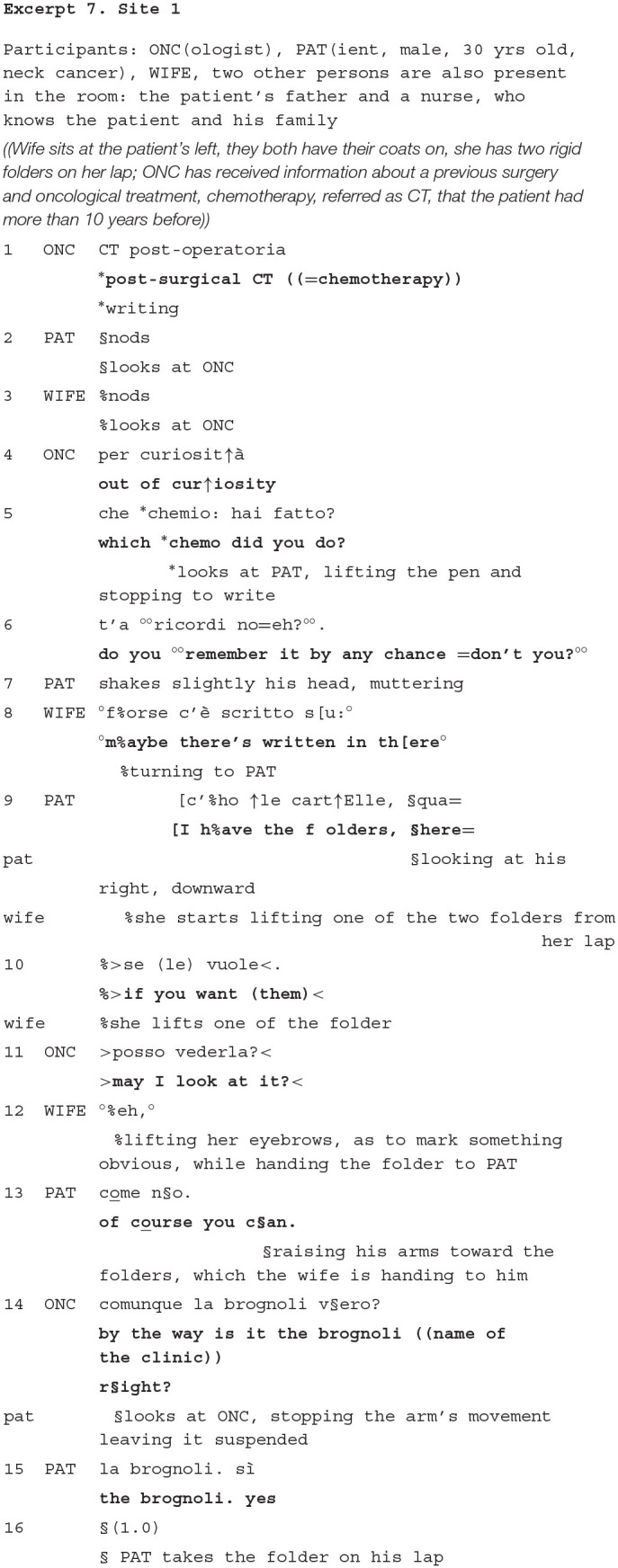


As we have observed in other visits of our corpus, the oncologist oralizes the information he writes (here, line 1), this way offering it at the scrutiny and confirmation of the patient (Sterponi et al., 2017; Fatigante et al., 2020). Following the oncologist's turn, the patient and companion nod concurrently, thus exhibiting themselves as having equal access to the information questioned and, further, to be equally legitimated to confirm it. In response to the oncologist's question whether the patient remembers the type of chemotherapy he did (several years before), the patient communicates that he does not remember. The patient's non-verbal token—the head shake (line 7)- undoubtedly conveys a clear reply, which would put to a close the inquiry. However, the patient's wife comes in the conversation (line 8) indicating the chance that what the doctor is looking for might be found in the written documents, contained in one (of the two) rigid, plastic folders which she carries on her lap.

Note that the companion whispers to the patient, and shows immediate proneness to hand the folders to him, but she never mobilizes (Stivers and Rossano, 2010) the patient's gaze to herself, nor she pursues in any way that he replies *to her*. That is, the companion's turn is crafted as an interstitial move and it is not expected by her, nor it is responded to by the patient, as a first part of an adjacency pair. Rather, it works as something that help the adjacency pair of question-answer between the doctor and the patient to continue smoothly. The patient, in turn, does not even wait for the end of her wife's turn to address a proposal to the doctor. The patient takes the wife's suggestion as a chance to provide the doctor with material evidences (which he refers as “his own,” despite the fact that he did not mention them before nor he noticed them as currently useful) from which the doctor himself can draw the information. So doing, the interaction flow between the doctor and the patient is never halted. Both the patient and the wife contribute to this. Correspondingly to the patient's proposal at line 9, and before any solicitation from him, the wife lifts the folders from her lap and starts handing one of them to him. Her move also anticipates the explicit request by the doctor, which she seems to assess as somewhat expected (line 12). It is also noteworthy to observe that, as the oncologist recruits the patient again to verbal interaction, the patient pauses the action he was entertaining with his wife (bringing the documents that she is handling to him), and makes also the wife wait. In this sense, the companion's move adjusts and closely follows the flow of interaction between the patient and the doctor as it goes.

Summing up, the contribution of the patient's companion here was essential, given that she uniquely made material surroundings, which might go “unnoticed” to the patient, available to both him and, ultimately, to the oncologist.

On the other hand, her contribution did not disrupt in any way the patient's interaction with the doctor.

We show another example that displays, however in a different fashion, how the companion is concerned that the patient properly responds to the doctor's questions and expectations.

In the excerpt 7, the patient (70 years old) is handling the relevant documentation regarding the surgery of the tumor to the doctor, and the doctor is transcribing the information in the medical record. The patient and her husband are monitoring the doctor's writing, looking at his pen on the sheet, remaining silent, while the doctor is oralizing (Sterponi et al., 2017). The doctor stops, quizzing about an incongruence in the chronological order of the documents.


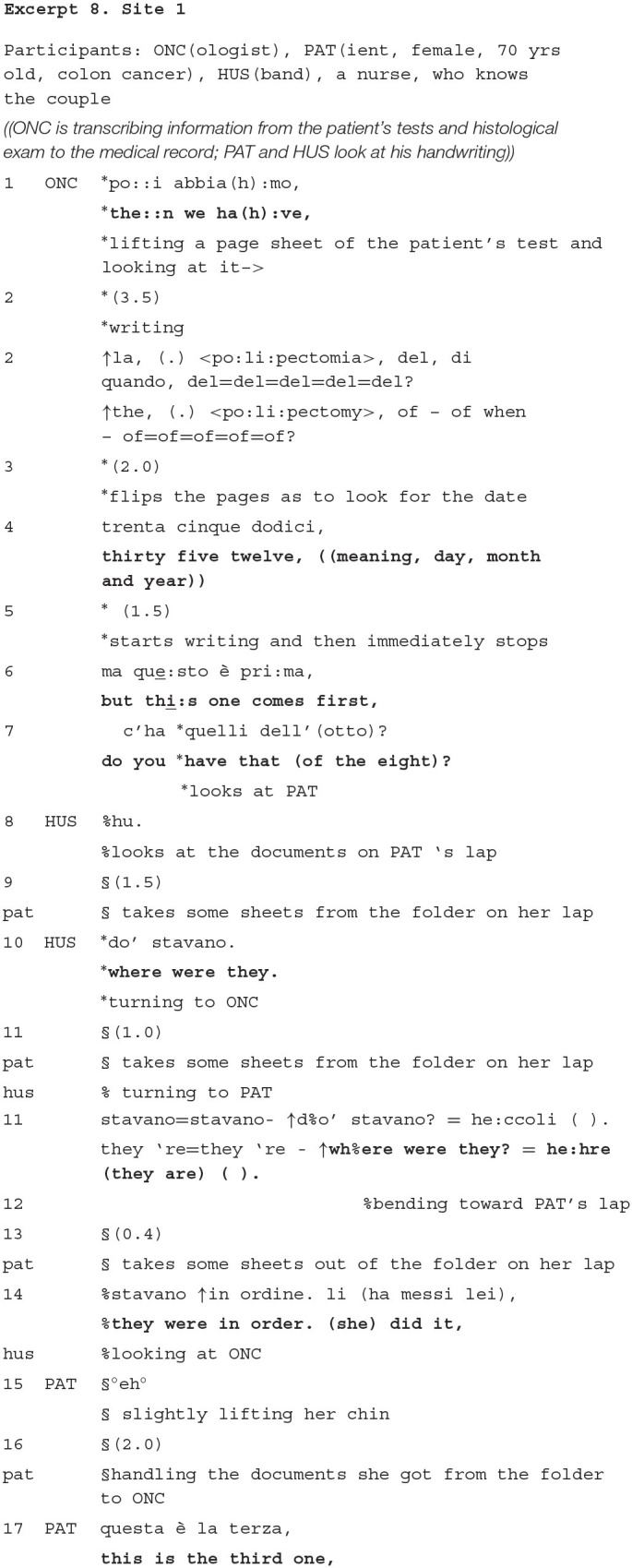


As we have already seen in the previous excerpt, the patient and her companion orient to the doctor's—sometimes long-activity of reading and transcribing remaining silent and gaze—following the oncologist's gestures throughout the writing. When the oncologist raises the problem about the incongruent order of the documents, the companion follows: first, uttering a change of state token (*hu*, line 8), i.e., an item that marks the acknowledgment of a certain piece of information as new and unexpected (Heritage, 1984b); then, he asks where the (missing) documents are.

In both ways, the patient's husband makes explicit his surprise with the current state of affairs, something, which makes him siding with the doctor's stance. Thorough his question, he looks in the direction of the patient's lap. So doing, it remains unclear whether he takes the wife as accountable for having messed up the documents or, more likely, he only utters the question as a generic marker of disappointment, which casts him as fully aware of the gap in expectations now having surfaced. His attempts would, then, work as to account for the poor performance that his wife and him are giving to the doctor. On the other hand, the wife does not visibly express such a preoccupation “about face” (Goffman, 1959) and provides no justification for the delay she eventually hands the document to the doctor (line 17). This and other similar instances show the companions' work in making relevant (either marking or, repairing) gaps in the patient's conduct, that is normatively expected in this context. It could also be that, being them more “free” from the pressure of both interacting with the doctor and being less emotionally loaded for the matters discussed, the companions can be more alert and they can also be more sensitive and available to exhibit a repair work of issues of face and performance.

### Brokering the Doctor's Explanations in Treatment Recommendation

The treatment recommendation is a particularly crucial stage of the visit: it incorporates the main institutional objective of this medical encounter, i.e., delivering a treatment plan for the patient, and it implies relevant efforts at both informational and emotional level for both parties at interaction. ASCO (American Society of Clinical Oncology) guidelines recommend that doctors describe all treatment options available, telling the patient the benefits and burdens of treatment and enabling the patients to understand and weigh them in order to engage in decision making (Gilligan et al., 2017). At the same time, previous studies have demonstrated how doctors are aware of the fears that patients have with regards certain therapies (mainly, chemotherapy) (Mulders et al., 2008; Davies and Yeoh, 2012). This infuses the work of providing information with a commitment to reassure them and lessening these fears, in the service of providing the patients with the most promising option for them (Sterponi et al., 2017; Fatigante et al., 2020). Studies indicate that companions, particularly if family members, play a relevant role in this stage, getting involved in decision making (Albrecht et al., 2010).

During the treatment recommendation stage, the patient is mostly oriented to listening to the oncologist's explanations that account for the different treatment options. Frequent nodding, production of continuers, acknowledgment markers (e.g., *sure, right*…) are among her/his most frequent contributions. Companions perform a similar work, but also, sometimes engage in more extended turns, that elaborate the doctor's explanation and facilitate doctor and patient's understanding of the (complex) information discussed.

In example 8, the doctor is anticipating to the patient the probable reason for why the surgeon (who has not yet operated the patient) has referred her to him. This implies the chance to reduce the size of the tumor by recommending the patient to undergo chemotherapy (so-called neo -adjuvant therapy) prior to the surgery. The explanation is not fully grasped by the patient, whereas, it is taken by the husband, who adds further material in order for the patient to understand.


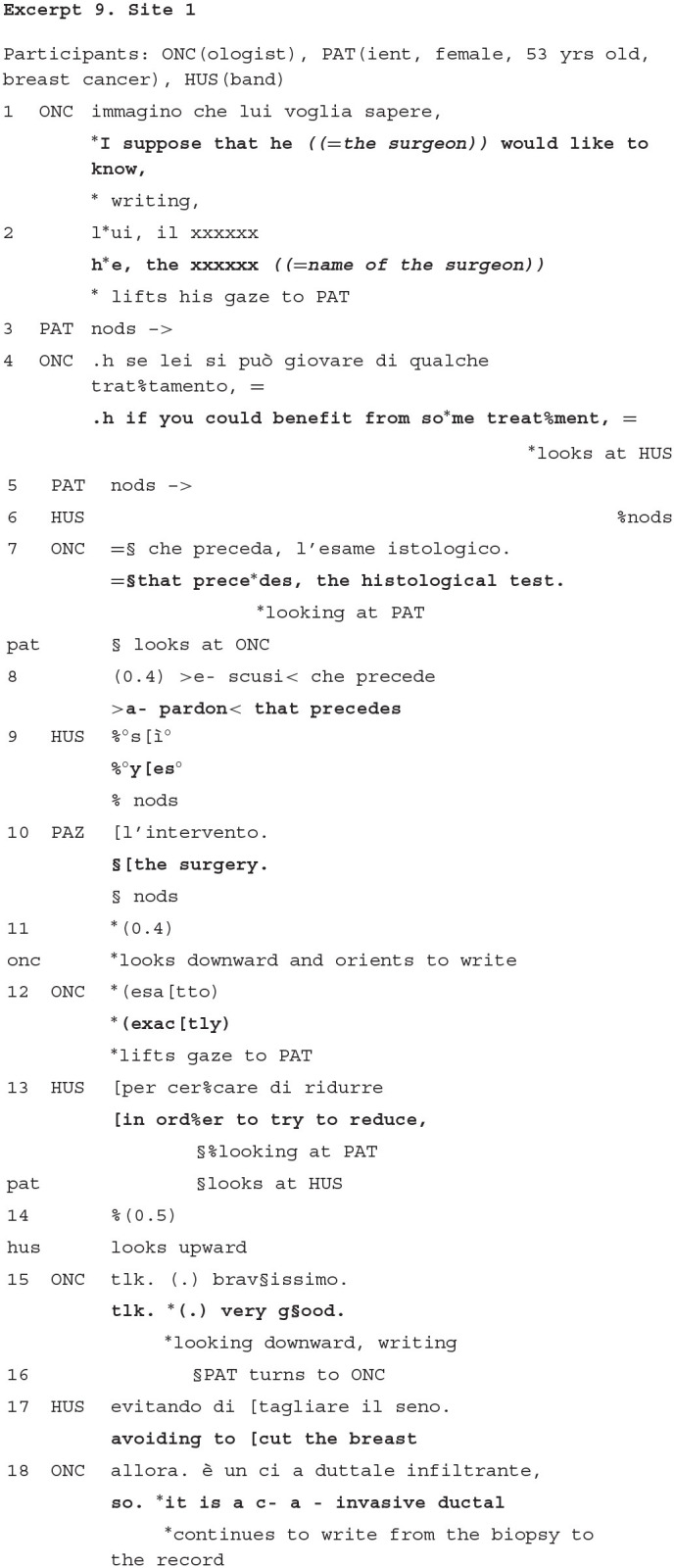


Until line 12, the companion and the patient are aligned as listeners of the doctor's turn, and they show to attend to the agenda of activities displayed by the doctor's both verbal instructions and physical actions, such as, writing. At line 13, the companion self-selects and adds original, medically relevant information, useful for brokering the patient's understanding with regards the benefit of undergoing a treatment before the surgery. This, together with the positive assessment he receives by the doctor, reveals that he has some knowledge regarding the conventional paths followed in the case of an oncological illness (he mentions, in another part of the visit, that his father had cancer and died for it). It is of interest for our discussion, that husband gazes at the patient at the beginning of his turn, addressing her as addressee of his talk. Immediately after, though (line 15), he looks upward, as if disengaging from this role of informant, thus rapidly clearing up the opportunity to engage in an open interaction with the patient, which would compete to the explanatory activity led by the doctor until now.

The example, then, manifests another evidence on how the companion works in a strictly contingent and situated manner with the local ongoing activity. By making sure that the patient understands the rationale of the oncological actions, he maintains orientation to an interstitial participation in placing his formulation at the margin of the official interaction between the doctor and the patient.

Both verbal and multimodal aspects concurrent to the delivery of the companion's turns attempt to convey a “double affiliation:” respectively, with the doctor, who is still acknowledged the institutional authority, and with the patient, who is helped to understand the implication of the oncologist's talk.

Excerpt 10 also displays how the companion's (here again, represented by the husband) contribution, although minimal, may be heard as overtly affiliating with the oncologist, and supporting the specific activity, i.e., the delivery of a treatment recommendation, that is relevant at this moment in the visit. The excerpt is taken from data in Site 2; the resident has just delivered to the patient the proposal of chemotherapy as the most advantageous treatment for her (lines not reported), and summarizes here the reason that supports such a proposal.


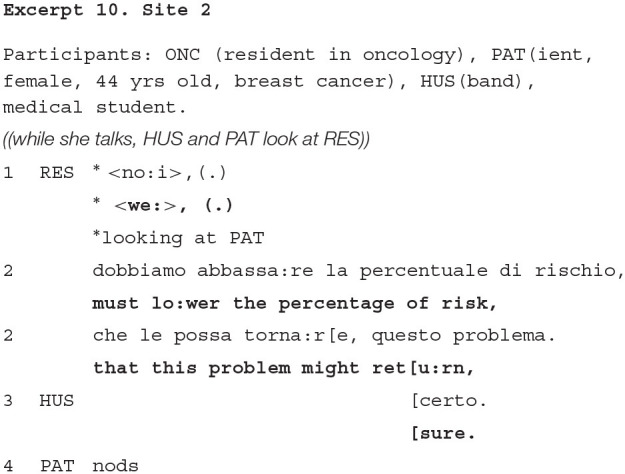


Both the patient and the companion display their availability as recipients of the doctor's explanation and recommendation, in that they maintain a sustained look to the resident throughout her formulation. Yet, the companion does more than that, producing a clear assessment (“sure,” also meaning “that's right”) of the oncologist's formulation (line 3). By making an assessment of the doctor's pronouncement, he signals that he embodies the oncologist's perspective (Goodwin and Goodwin, 1987) and supports the validity of the doctor's treatment recommendation. So doing, the husband's contribution obtains to sequentially prepare and shape the patient's orientation to eventually agree with the content of the formulation (line 4).

Summarizing, through many different cases and patients with different ages and cancer types, we have found that companions in this stage appear to support the importance for the patient to understand and accept the treatment proposed. Therefore, their contribution may be heard as essential for soliciting the patient to embrace a decision. At the same time, the in-depth analyses of how they intervene add to the main finding of the study, for which they tend to not “grab the spotlight” upon themselves and not interrupt the official interaction between the patient and the doctor.

### Introducing Practical Matters in the Outline of Future Actions Stage

We discuss one example from the stage we labeled “Outline of future actions” that occurs after the main and most delicate business of this kind of encounter (the discussion of the treatment option) has been done. First, we have to highlight that, in this stage, the companions are consistently observed as being more active in recruiting the doctor as their own addressee, asking him questions about practical concerns related to the therapy such as, its duration and the ways in which it will be delivered. Questions about the side effects of the therapy are also voiced by companions more than by patients.^1^ A study by Eggly et al. (2006), which coded both the companions' and patients' questioning behavior, also found that the former more often raised issues about the management and logistics of treatment procedures. The companions' contribution appears as of utmost relevance, due to the fact that patients in first visits may be overwhelmed by the quantity and complexity of information they get in the previous stages, which they also need to process while they are presumably in a state of anxiety and concern (Annunziata and Muzzatti, 2012; Bronner et al., 2018). If we look at these instances, we find that the companion's question is taken by the doctor as a chance to deliver the information to the patient also. See at this regards example 11:


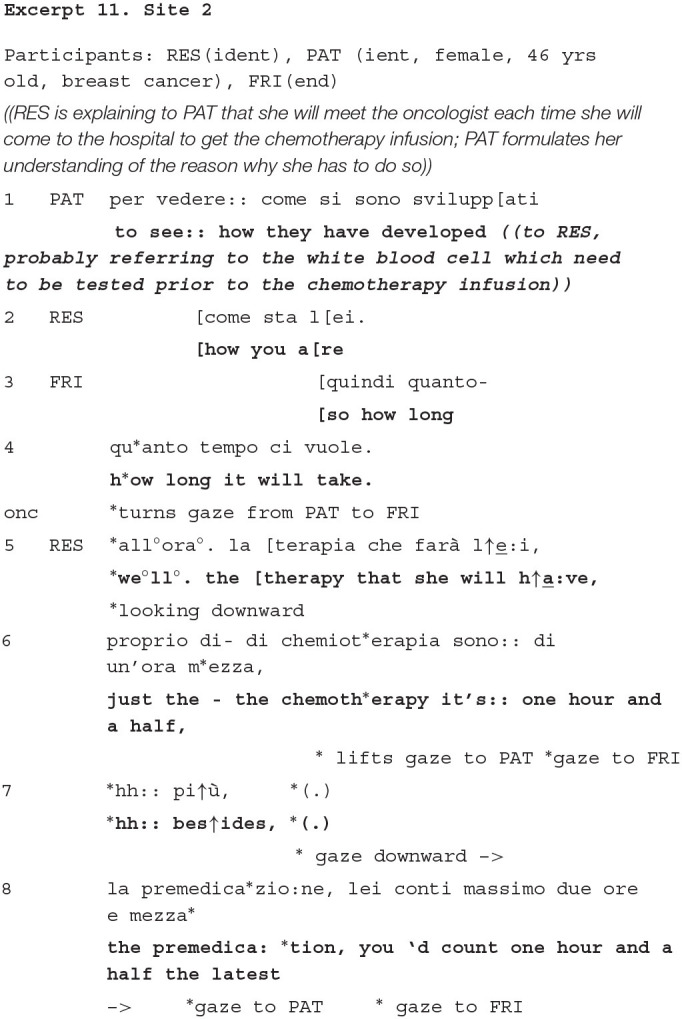


The companion here self-selects as the resident's previous turn approaches completion and she asks about the duration of each infusion. In responding to her, the resident removes her gaze and starts looking downward, as if planning her response. When she lifts her gaze, she looks at the patient and not at the companion, and she only shifts gaze to the companion when she tells the measure (one hour and a half, line 6), which was exactly what the companion asked. Further, as she expands her response, adding details, she explicitly selects the patient (line 8), turning again to the companion only at the end of the turn, and correspondingly to the mention, again, of the exact length.

The next and final example shows a similar case. Here, the patient has shown her distress in apprehending that she will get chemotherapy. The resident is adding information about the way she will get the chemotherapy infusion and she tells the patient that she will be implanted a device, the portacath (Port), which will give access to veins for regular administration of the drug.


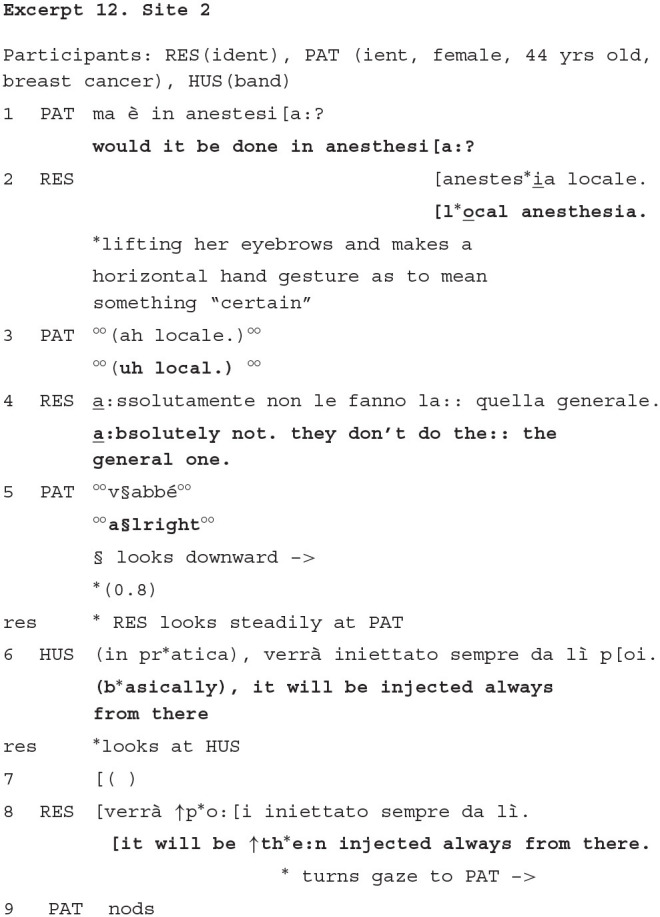


Here again, the companion self-selects to ask a question to the resident. Though, early in the construction of her response, the resident shifts gaze to the patient (line 8) and never returns it to the companion, although the topic is maintained for few lines more (lines not reported). This and other examples make clear that, even in cases where the companions clearly constrain doctors to attend to them as primary interlocutors (as in question-answer pairs), the sequential and multimodal construction of the doctor's answer is crafted in such a way, as to maintain companions, and not the patients, as “audience” to the doctor's response.

### “Speaking As” the Patient: An Example From the Physical Examination

Among our excerpts, we found one single instance in which the companion casts the patient in the third person, apparently speaking on her behalf.

We first provide the context of the sequence. The exchange takes place as the visit approaches conclusion (minute 33′47″, where the overall length of the visit is 40′22″). In this visit, the patient meets this oncologist for the first time. However, she had already experienced cancer long before. Prior to this sequence, the patient has made repeated attempts to be reassured by the oncologist, regarding a specific concern, i.e., her fear that a certain value, called CA 125 (amount of the protein cancer antigen 125 in the blood), can grow: CA 125 is sometimes used as a marker of cancer recurrence in patients with ovarian cancer, like the patient in this visit. The doctor has engaged in a long explanation about this, where he told the patient that an increase of CA 125 is also observable in a variety of other, non-oncological and non-risky conditions. For this reason, he recommended the patient to stop persisting in what he called an “obsessive” measuring of the marker (the patient also described herself as “crazy” about checking the marker), ironically stating that “otherwise she would spend all her life to check the CA 125.” At this point, the doctor invites the patient to undress in order to proceed to the physical examination. Throughout the doctor's explanation that has occurred up to now, which has also included that the doctor has gazed to both the patient and the companion, the companion has remained silent. He only takes turn as the doctor asks the patient to proceed to the physical examination:


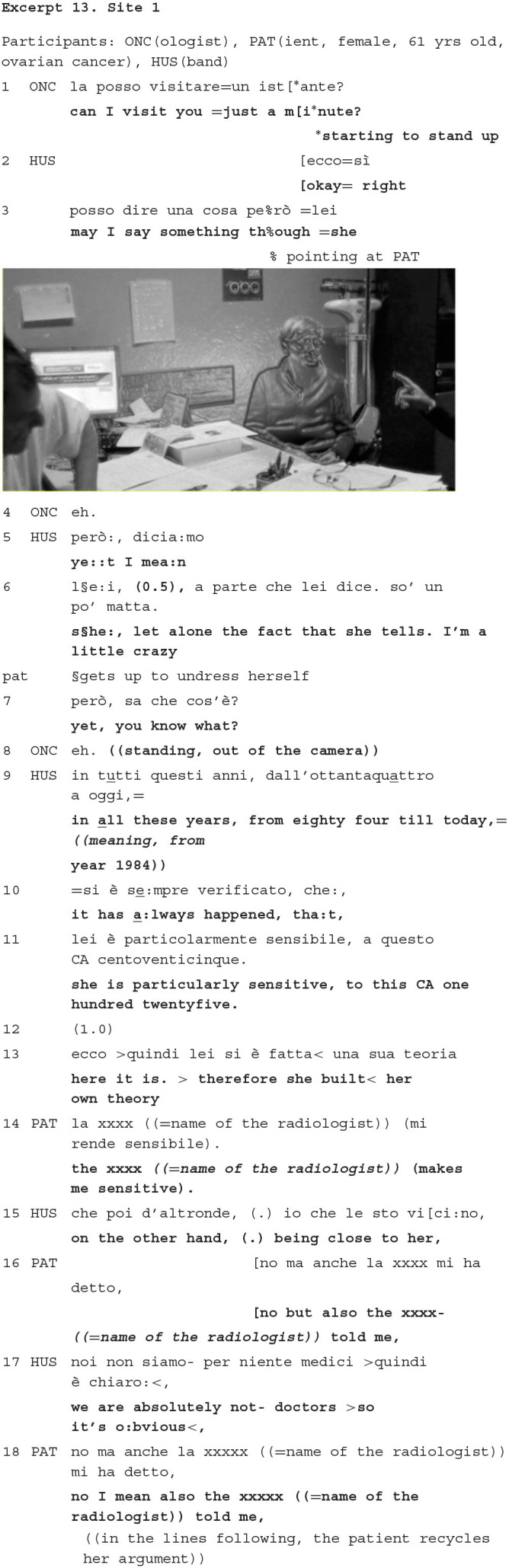


We need first recall that the oncologist has already read the series of medical diagnostic documents that the patient has brought to the visit, and formulated his opinion to the patient. Therefore, we can consider that the stages of Cancer Diagnostic Assessment and Treatment Recommendation (here, involving an advice for a “wait -and -see” strategy) have already occurred. The companion's initiative at line 2 is perfectly timed: he enters in correspondence to the TRP (Transitional Relevance Point; Sacks et al., 1974) but also at the juncture point of the activity transition, marked by the oncologist's question and his postural change (he stands up).

At the same time, his intervention poses a problem, in that he recruits the oncologist's attention, in a moment where the oncologist is just leaving from the attentional visual field (the desk) available to interact face to face with the husband. In this sense, the companion's move could be sensed as inappropriate and untimely. The companion anticipates the unwelcomed consequences of his possibly inappropriate move by a pre-sequence (line 1, see Schegloff, 1980, 2007), a polite formulation which works in order to obtain a go-ahead response from the doctor, prior to the telling; by doing so, the companion also shows his reflexive awareness that he might be not fully entitled, in this context, to address original concerns. Last but not the least aspect we want to emphasize, the husband points at his wife with his finger, this way maintaining her—who was also just about to stand up- as involved in the telling. His pointing action solicits, in turn, that the wife turns gaze at him and awaits (until line 6, when she stands up). In the turns that follow (lines 7–13), the husband advocates the patient's reasons to voice her concerns to the doctor, regarding the CA125 marker. Noteworthy, the companion does not support the patient's concern that the marker can indicate a progression of the illness. This argument has been clearly rejected by the scientific explanation that the doctor has provided to her in the lines prior to this sequence (not reported). Rather, the husband supports the patient's entitlement to develop her own theory, due to the evidences that she gathered through time (line 8), on a period, upon which she can uniquely claim experience. In so doing, the husband engages in repair work as regards the potential face loss (Goffman, 1959) that the wife had experienced in the discussion with the doctor; he does so, by strengthening the epistemic bases of her claims which, although typologically different from that of the doctors (see the disclaimer at line 17, “*we are not doctors”*), are notwithstanding viable to shape a personal explanatory theory of the problem. Note that the husband juggles from emphasizing the wife's oddness (line 6), this way apparently siding with the doctor against her argument, to validating the wife's evidences, by putting them in historical context. In these terms, what the husband does could be righteously captured by what Mazer et al. (2014) call as “speaking *about”* the patient. Accordingly to that categorization, here the husband's “role” would be that of an external observer, who infers “the patient's cognitive processes” (she built her own theory, line 13) from an external and independent assessment. How such a categorization is vulnerable to instability is, though, clear as we come to line 15 immediately following, when the husband claims to be “close to her” (line 15), and thus invokes his own position as co-experiencer of the patient (Mazer et al., 2014).

However, the husband maintains a certain ambiguity (also surfacing in his vague formulations, as in “she is particularly sensitive” or in the elliptical sentences at line 15) as to whether he is embracing the patient's view or just attempting to repair her face loss, his intervention helps indeed the patient to recycle her argument: that is, the patient takes advantage of the companion's turn, to invoke the radiologist's opinion (lines 14, 16, 18) and build a new confrontational arena for the oncologist to respond again to her issue.

That the oncologist opinion here is called into question again is also demonstrated by the husband's disclaimer “we are not doctors” (line 17), that *mitigates* (Caffi, 1999) the validity of the propositional content alluded by his intervention.

With this final example, we showed how, during the first oncological visit, taking over the patient's position and *speaking* for or about her is a complex endeavor for the companion. Particularly, and whether or not it is intended to support or diminish the patient's agency, the companion's move displays as never independent from the sequential context and the existing constraints of the participant framework that are active in the visit at any specific moment. We add, as a final point, that the companion takes active initiative as the main verbal flow of interaction the patient and the doctor is momentarily suspended, in favor of an activity which, involving the body inspection of the patient, is managed as separate and self-bounded. This adds as an ultimate evidence of the companions' attention to act at the margins of the main, official discourse activities or at most, at the interstices between activities.

## Discussion

In this paper, we conducted a close analysis of how the companions of cancer patients who are present at the first oncological visit contributed to the accomplishment of the visit and its different aims. Our results add and complement those, reported from previous studies on the topic, which indicate that, overall, companions present in the visit *facilitate* the communication between the doctor and the patient (Laidsaar-Powell et al., 2016). Our analyses confirm this result in the specific oncological setting examined, and they show how the companions facilitate doctor-patient communication in rather specialized ways, i.e., by supporting the specific institutional aims and activities related to each different *stage* of the visit.

Summarizing, and precisely focusing on the temporal and sequential arrangement of the verbal and multimodal resources that are employed by all participants in the visit, our analyses uncover some regular features.

Firstly, we showed that the companions design their turns as to acknowledge and preserve the doctor- patient talk as the main and official course of interaction since the very beginning (excerpts 1–2). This is primarily revealed by instances, where the companions deliver their turns—however sometimes essential to the accomplishment of specific activities (excerpts 3, 4, and 7)—in a reduced volume or, where they “mask” or lessen the visibility of their actions in such a way, as to not recruit the participants' overt attention. Companions also allowed themselves to openly enter the conversational floor and publicly recruit all other co-participants' attention, but this only happened at certain moments in the visit, that clearly come *after* the topical business of the visit (i.e., diagnostic explanations and treatment recommendation) has been discussed (excerpts 11–13).

This maintains the patient as the most responsible and legitimate agent in the interaction and allows that the patient's interaction with the oncologist runs smoothly. The examination of multimodal resources was particularly beneficial to the investigation of this preference. We, thus, discovered that, when the companions speak on behalf of the patient, they finely negotiate with the patient, by means of constant gaze monitoring and gaze shifts, their own entitlement to speak (excerpts 5–6). We also noted that doctors responded to companions' either verbal and non-verbal turns by “rushing through” the interaction with them, and returning rapidly to the patients, as when they reply to the companion's question addressing the patient or the companion-patient pair together (excerpts 3, 6). By doing this, they honor the patient's status as their proper interlocutor, at all stages of the interaction and informational flow.

A second strong finding is that companions show to attend to the particular goal orientation (Drew and Heritage, 1992) of each *stage* of the visit. Gaze shifts, gestures, modulation of the tone of voice (e.g., whispering), pausing, interstitial placement of actions such as, passing documents silently, or muttering, help companions to attune to the specific pragmatic constraints regulating the transitions between stages and activities (Mondada, 2006; Deppermann et al., 2010). By including all these multimodal resources, the analyses pointed out how the companions revised and strengthened the patient's report during the Anamnesis and Problem presentation (excerpts 3–6); they helped in the provision of documents to the doctor and monitored carefully both the patient and the doctor's conduct during the screening of the patient's records during the Cancer Assessment stage (excerpts 7–8); they supported the oncologist's formulation and bridged doctor-patient mutual understanding in stages, where the doctor delivered explanations and instructions during the Treatment Recommendation stage (excerpts 9–10); they postponed their more active interventions (e.g, questions) to the end of the visit, orienting to the institutional goal of warranting the patient the more information as possible about how to practically manage the therapy once the visit is over (excerpts 11–12).

Thirdly, contrary to what identified by previous studies on the topic (cf. Street and Gordon, 2008; Jansen et al., 2010), our research does not report enough evidences of the companion's provision of “emotional support” to the cancer patient. That is, we did not observe explicit attempts by the companions to provide reassurance or comfort to the patient, nor instances in which they explicitly check with the patient, or voice to the doctor, the patient's feelings. We may not exclude that companions in our corpus did so, later and outside the consultation room; yet, it appears that, throughout the visit, companions prioritize those contributions, which are beneficial (essential in some cases) to the accomplishment of the instrumental tasks embedded in the first oncological visit, rather than to soothe, or formulate, the patients' possible discomfort or anxiety. On the other hand, we also take a cautious stance with regards the term emotional, in that, as Ruusuvuori (2013: 230) maintains, identifying how—and by whom-emotions are dealt or expressed in talk is a challenging task. Keeping close to the evidential ground of our transcripts, we have seen that the companions displayed affiliation toward the doctor (excerpt 1 line 7, excerpt 3 line 17) and sometimes appeared to be openly judgmental of the patient's performance (excerpt 6 line 5, excerpt 13 line 6); yet, when this happened, they managed, *via* gaze and sequential placement of their turns (such as, withdrawing and waiting that the patient takes her turn or validate their responses, removing gaze from the participants' and leaving that the doctor-patient interaction is resumed, attempting to get the patient involved again etc.) to restore and balance patient's entitlement to participate, thus affiliating to her/his status as primary teller of her/his experience. Excerpt 13, in particular, showed a case in which the companion does “repair work” (Goffman, 1959) toward the patient's *face*, by supporting her entitlement to have a say upon her own experience. Despite the husband points to the patient's “sensitivity,” it is not emotions that are made relevant but, rather, the patient's opportunity (which she takes advantage of) to recycle her argument and ground it onto more authoritative bases (i.e., the support from another doctor).

That is, however the husband's move might be intended to assist the patient in her emotions, the development of the sequence showed how it encountered a different treatment and interpretation. It may be that these results are constrained by the particular institutional and interactional context of this kind of visit: this is an event, occurring between people who are mutually strangers, primarily designed to deliver information and instructions for the patient about what treatment to begin and how to perform that treatment (Sterponi et al., 2017; Fatigante et al., 2020). Emphasis is placed upon the need that the patient understands—and agrees with—the epistemic bases (i.e., the explanation of benefits and risks), which favor certain treatment options instead of others (see also Costello and Roberts, 2001; Collins et al., 2005; Gill, 2019; Tate and Rimel, 2020). The discussion of psychosocial concerns—which may also threaten the patient's availability to agree with the proposal—is at most postponed to the accomplishment of the main task of the visit, and they are certainly not dedicated a proper and specific stage.

A final point we discuss in relation to the reviewed literature, is that our study does not support findings, which indicate that the presence of the companion can generate tensions with the patient, due to marginalization or censorship of the patient's voice (cf. Mazer et al., 2014). It might be that such tensions arise more in contexts, where the patient is elderly or more vulnerable than the patients in our corpus. However, we also add that we posed a particular attention to avoid assigning a unilateral judgment over the companions who took turns on behalf of the patient. As shown, the in-depth examination of the sequential multi-party arrangements of participants' turns demonstrate that the companions' actions are in fact always the result of a negotiation with the patients, who are not passive even when they remain silent (Heath, 1986). Furthermore, the doctors in our corpus ultimately appeared to refer to patients, or, at most to the companion-patient dyad as their ultimate addressee.

We conclude, by underlining that our study provide nuances to the role categorizations ascribed to companions by coding schema in previous studies (Ellingson, 2002; Street and Gordon, 2008; Del Piccolo et al., 2014; Mazer et al., 2014). In contrast to the emphasis on verbal conduct as primary means of interaction, and the focus on individual behavior implied by coding schema, the analytical procedure we have chosen closely followed the participants' actions in the local time and place they are produced (the conversational sequence). Within this perspective, we showed that the companions' *roles*, rather stable positions enacted by the individual actor, are instead highly mobile; they are tied to the specific contingencies occurring within the visit stages and activities, and they are the contextualized results of complex temporal, sequential, multimodal and multiparty arrangements of all participants' actions (these including minimal visual tokens such as eye movements and head/body inclines, or aural tokens such as sighs and hummings, rarely examined by coding systems).

The evidence for which the companions are able, by even small and interstitial actions, to support the accomplishment of the oncological visit's institutional aims and activities, emphasizes the potential relevance of their contribution in affecting both the visit's outcomes and the participants' satisfaction of their communication.

Our findings suggests the need to broaden the concept of the doctor-patient alliance into that of a doctor-patient-companion alliance. Medical education, and interventions to implement patient's and companion/caregiver's literacy in oncology, have to consider the multiparty nature of the oncological communication. On the other hand, we demonstrated that the richness of doctor-patient-companion communication can be utmost revealed by analytic tools, such as multimodal video analysis of conversations, that shows carefully and preserves the local configuration of actions assembled moment by moment by participants (Mondada, 2006).

## Data Availability Statement

The data analyzed in this study is subject to the following licenses/restrictions: the data that support the findings of this study are not publicly available due to privacy restrictions. Requests can be addressed to the authors, as to only share transcripts of the interactions (with anonymized references). Requests to access these datasets should be directed to Cristina Zucchermaglio, cristina.zucchermaglio@uniroma1.it.

## Ethics Statement

The studies involving human participants were reviewed and approved by Ethical Committee of Policlinico Umberto I, Ethical Committee of Department of Social and Developmental Psychology, Sapienza University of Rome and ASL (Local Health System) RM A, Rome. The patients/participants provided their written informed consent to participate in this study. Written informed consent was obtained from the individual(s) for the publication of any potentially identifiable images or data included in this article.

## Author Contributions

MF worked at the first draft of the results, which were revised by CZ first and FA. FA contributed to the writing of the introduction. CZ contributed to the writing of the Discussion and Implications. All authors contributed to the data collection, the concept and design of the study, the planning of the contribution in its aims and methodology, and contributed to the collection and discussion of the analyses.

## Conflict of Interest

The authors declare that the research was conducted in the absence of any commercial or financial relationships that could be construed as a potential conflict of interest.

## Footnotes

^1^On a subcorpus of 22 visits examined for other purposes, we have done a preliminary examination of the number of the companions' questions in the different stages of the visit, finding that the stage “Outline of future actions” contains the highest number of questions than in any other stage: 116 questions vs. 34 and 24 in, respectively, the stages of Treatment recommendation and the Cancer diagnostic assessment.

## Appendix A

### Transcription Conventions (Jefferson et al., 1987; Mondada, 2018)

**Table:**

.	falling, or final, intonation contour, not necessarily the end of a sentence
?	rising intonation, not necessarily a question
,	ascending and “continuing” intonation, not necessarily a clause boundary
[	indicates where the overlap begins
]	indicates where the overlap closes
-	a cut-off, abrupt halting of sound or a word
(1.2)	silence in tenths of a second
(.)	a ‘micropause', less than 2/10 of a second
=	latching, meaning no break or delay between the words thereby connected
>word <	an utterance or its portion is delivered at a pace noticeably quicker than surrounding talk
< word>	an utterance or its portion is delivered at a pace noticeably slower than surrounding talk
:::	stretching of the preceding sound, proportional to the number of colons
↑↓	marked rising and falling shifts in intonation
word	stress or emphasis on the underlined item
WOrd	indicates that an utterance or its portion is louder than the rest of the talk
°word°	indicates a passage of talk noticeably softer than surrounding talk
.hh	audible in-breath
hh	audible out-breath
(word)	indicates uncertainty on the transcriber's part
( )	indicates that something is being said, but no understanding could be achieved
*((word))*	enclose description of conduct or, context

### Notation for Visible Actions

**Table:**

*	points where the doctor's visible behavior (e.g., a nod) starts or ends
§	points where the patient's visible behavior (e.g., a nod) starts or ends
%	points where the companion's visible behavior (e.g., a nod) starts or ends
—>	the action described continues across subsequent lines
